# A comparison in knee flexor and extensor strength following ACL reconstruction in international, male soccer players receiving patellar tendon or hamstrings grafts

**DOI:** 10.5114/biolsport.2024.129471

**Published:** 2023-07-19

**Authors:** Andreja Milutinović, Vladimir Jakovljević, Milinko Dabović, Aaron T. Scanlan, Dragan Radovanović, Aleksandra Orlova, Emilija Stojanović

**Affiliations:** 1 Department of Physiology, Faculty of Medical Sciences, University of Kragujevac, Kragujevac, Serbia; 2 Department of Human Pathology, 1^st^ Moscow State Medical University IM Sechenov, Moscow, Russian Federation; 3 Faculty of Sport and Physical Education, University of Belgrade, Belgrade, Serbia; 4 School of Health, Medical and Applied Sciences, Central Queensland University, Rockhampton, Australia; 5 Faculty of Sport and Physical Education, University of Niš, Niš, Serbia

**Keywords:** Football, Injury, Rehabilitation, Ligament tears, ACL

## Abstract

The aim of this study was to compare knee extensor and flexor strength recovery following anterior cruciate ligament (ACL) reconstruction between bone-patellar tendon-bone (BPTB) and hamstring tendon (HT) grafts in international male soccer players undergoing comparable 6-month rehabilitation programmes. Seventeen players underwent ACL reconstruction with either an autogenous BPTB graft or HT graft. Knee extensor and flexor peak torques were measured at 3 months and 6 months in the injured and contralateral legs following surgery using isokinetic dynamometry. The *moderate–large* asymmetries in knee extensor peak torque between legs at 3 months across graft types (BPTB: p = 0.002, g = -0.94; HT: p = 0.02, g = -0.55) were reduced to *trivial* asymmetries at 6 months (BPTB: p = 0.30, g = -0.19; HT: p = 0.40, g = -0.16), with a non-significant difference in limb symmetry index (LSI) between grafts at 6 months (p = 0.62, g = -0.24). Similarly, *moderate–large* asymmetries in knee flexor peak torque between legs at 3 months across graft types (BPTB: p = 0.13, g = -0.50; HT: p = 0.01, g = -0.97) were reduced to *trivial-small* asymmetries at 6 months (BPTB: p = 0.25, g = 0.18; HT: p = 0.01, g = -0.47); however, a superior LSI was evident with BPTB compared to HT grafts at 6 months (p = 0.007, g = 1.43, *large*). Strength and conditioning professionals working with soccer players who are rehabilitating from ACL reconstruction after receiving a HT graft should give adequate attention to delivering suitable hamstring exercises that ensure optimal strength restoration.

## INTRODUCTION

Soccer is the most popular team sport worldwide, with more than 275 million active players [1]. Soccer players perform a range of intense movements, such as accelerations, decelerations, jumps, kicks, changes in direction, and tackles during training and matches. In turn, many scenarios surrounding these movements including being tackled or tackling an opponent, regaining balance following kicking, and landing from jumping have been observed to take place when professional soccer players sustain an anterior cruciate ligament (ACL) injury [2]. The incidence rate of ACL injury in European professional male soccer players has been reported to range from 0.04 to 0.06 per 1000 h of combined training and match exposure [3–5]. In turn, ACL injuries are among the most devastating injuries encountered by professional soccer players, requiring extensive rehabilitation involving long lay-off times from training and competition [6]. The return time to competition of professional soccer players is particularly important given the economic and performance implications for professional soccer teams accompanying the absence of players due to ACL injuries.

A complete or partial ACL rupture can lead to recurrent instability, meniscus tears, chronic pain, and osteoarthritis [7, 8]. To reduce the risk of further damage, arthroscopically assisted ACL reconstruction has become the most common method to repair a complete ACL rupture, in which an autograft (own tissue) or allograft (tissue taken from another person) replaces the torn ligament [9]. In turn, use of the central third of the patellar tendon (bone-patellar tendonbone [BPTB]) or use of the semitendinosus and gracilis tendons (hamstring tendons [HT]) are the most frequently used graft types for ACL reconstruction [10]. Although acceptable knee function and stability have been observed following ACL reconstruction using both graft types [11], they will each inherently impair the strength of muscles surrounding the knee, with deficits of nearly 50% reported at 4 weeks after ACL reconstruction [12, 13].

Although a slight predominance of one leg over the other is common in soccer players [14], inter-limb asymmetries in knee extensor and flexor strength of > 10% have been suggested to raise knee injury risk [15]. In this regard, adequate functional recovery following ACL reconstruction is accepted when strength in the injured leg in relation to the contralateral leg reaches a limb symmetry index (LSI) of ≥ 90% [16]. In contrast, inadequate knee extensor strength following ACL reconstruction yields greater rates and magnitudes of load transmission from distal to proximal segments of the leg to increase the risk of ACL graft re-rupture, contralateral knee injury, or premature degenerative changes in the repaired knee joint [2, 17]. Also, optimizing knee flexor strength is crucial when undergoing rehabilitation following ACL reconstruction given that this muscle group buffers shear forces by preventing anterior slide of the tibia relative to the femur [18]. Furthermore, hamstring activation stabilizes the knee in response to external varus and valgus loads [19]. Despite the importance of restoring knee extensor and flexor strength following ACL reconstruction, low rates of competitive [20] and professional [6] male soccer players with BPTB [6, 20] and HT grafts [6] have been reported to achieve the desired LSI of ≥ 90% at 6–9 months following surgery [20] or when returning to unrestricted play [6]. Nevertheless, there is a lack of data directly comparing knee extensor and flexor strength recovery between BPTB and HT autografts in professional male soccer players. Such a comparison is essential given that graft type selection for ACL reconstruction may influence muscle strength recovery and therefore the duration required for a safe return to competition [21].

While there has been a lack of research on this topic specifically in soccer players, comparisons in strength recovery following ACL reconstruction between different graft types have been conducted in recreationally active individuals [22–24], non-athletes [25], non-professional athletes [26–29], military cadets [30], and athletes competing at varying playing levels pooled together [31, 32]. In addition to varied activity and playing levels, the available data have largely been pooled across athletes competing in different sports [27, 28, 30] and across sexes [21–28, 30–32]. Although these studies provide important insight into strength recovery following ACL reconstruction, the existing evidence may not be transferable to professional male soccer players considering that higher strength capacities have been observed in players competing at this playing level compared to lower levels [33]. Furthermore, professional soccer players likely have greater access to wider resources (e.g., practitioner supervision, facilities, equipment) during the rehabilitation process compared to players competing at lower playing levels, aiding player recovery. Likewise, evidence stemming from pooled findings across players competing in different sports [27, 28, 30] and across sexes [21–28, 30–32] should not be simply applied to male soccer players, given the different knee extensor and flexor strength levels reported according to these factors [34] and varied approaches adopted in managing the rehabilitation process [16]. Therefore, examination of knee strength recovery following ACL reconstruction should be conducted strictly in professional male soccer players for greater specificity in evidence for application in this population.

Review of the existing evidence comparing knee extensor and flexor strength recovery following ACL reconstruction between BPTB and PT grafts reveals inconsistent findings [21–28, 30–32]. Specifically, some research has documented significantly inferior knee extensor strength with BPTB autografts compared to HT autografts (5 months–5 years following surgery) [21, 23–26], while other research reported non-significant differences in knee extensor strength between graft types (6 months–3 years following surgery) [22, 26–28, 30–32]. In contrast, studies have reported inferior knee flexor strength with HT autografts compared to BPTB autografts (5–29 months following surgery) [21, 22, 24, 26, 27, 32], while separate research demonstrated non-significant differences in knee flexor strength between graft types (6 months–5 years following surgery) [23, 25, 28, 30, 31]. Although professional male soccer teams are under immense pressure for players to return to unrestricted play within 6 months [35] following ACL reconstruction, there is a lack of data documenting recovery in knee extensor and flexor strength up to 6 months following surgery using BPTB and HT autografts. Therefore, the aim of this study was to quantify and compare knee extensor and flexor strength recovery following ACL reconstruction between BPTB and HT autografts in international male soccer players undergoing comparable 6-month rehabilitation programmes.

## MATERIALS AND METHODS

This study adopted a within- (inter-limb comparisons) and between-subject (BPTB vs. HT autografts) design to compare knee extensor and flexor strength recovery following ACL reconstruction in international male soccer players undergoing comparable 6-month rehabilitation programmes. Data were gathered at a private physiotherapy fitness centre Femur over a 4-year period until December 2022. All procedures were approved by the Human Research Ethics Committee of the Faculty of Medical Sciences at University of Kragujevac, in Serbia (01-11122) in accordance with the Helsinki Declaration.

### Subjects

Seventeen international male soccer players from 15 different teams with a primary diagnosis of ACL deficiency underwent arthroscopically assisted ACL reconstruction with either an autogenous BPTB graft (n = 8, age: 23.1 ± 4.0 years [range: 18–28 years], height: 182.1 ± 2.7 cm; body mass: 77.5 ± 3.5 kg) or HT graft (n = 9, age: 24.8 ± 4.9 years [range: 19–32 years], height: 182.7 ± 7.5 cm; body mass: 76.5 ± 7.7 kg). Only soccer players competing at the international level immediately prior to sustaining the ACL injury were recruited to ensure that an elite playing sample was examined [36]. Subjects were able to participate in the study if free from any significant meniscus lesions (only a partial meniscectomy at most), chondral damage (as assessed either from magnetic resonance imaging or by the orthopaedic surgeon at the time of surgery), previous ACL injury (in the injured or contralateral leg), and other musculoskeletal injuries that could negatively affect the results of the study. Accordingly, 4 of the 21 soccer players originally recruited were excluded, leaving 17 players (from Serbia, Croatia, Ukraine and Switzerland) participating in the study. All subjects provided written informed consent after the procedures of the study were explained, including the risks and benefits associated with participation.

### Procedures

Subjects were allocated to the BPTB or HT group based on the autograft technique used for their ACL reconstruction. Both the BPTB and HT grafts were secured on the femur and tibia using interference screws. In the BPTB group, the central third of the patellar tendon was harvested with a vertical incision by blocking the patellar and tibial bones. A standard longitudinal incision over the pes anserinus was used for harvesting the HT graft. The BPTB autograft was between 9 and 10 mm in diameter, while the HT autograft was between 7 and 9 mm in diameter. All operations were performed by the same orthopaedic surgeon who specialized in conducting both ACL reconstruction techniques. The technique was selected for each subject according to their own choice with input from the surgeon. Injuries to both the dominant and non-dominant legs were involved in the sample (dominant leg: n = 7; non-dominant leg: n = 10). All subjects underwent the same standardized 6-month rehabilitation programme at the same private physiotherapy fitness centre. Isokinetic knee (extension and flexion) muscle strength was measured at 3 months and 6 months following surgery during the rehabilitation process. All testing sessions were carried out in similar environmental conditions for all subjects (~22 °C and ~60% relative humidity) and at a similar time of day (09:00 to 11:00). A verbal explanation and demonstration of the testing procedures were given to each subject prior to each testing session. Subjects completed a standardized warm-up prior to the isokinetic strength tests, consisting of a 5-min moderateintensity exercise bout on a cycle ergometer (Group Cycle Ride, TechnoGym, Gambettola, Italy) [37, 38] followed by passive stretching exercises focused on the quadriceps, hamstrings, hip adductors, and calf muscles [39], as well as three submaximal knee extension and flexion movements for each leg at an angular speed of 60° · s^−1^. Stretching positions were held for short durations (15 s per muscle group) to avoid any subsequent negative impacts on performance [38] and ensure that proper body alignment was attained, involving subjects being in a comfortable and correct position that optimizes range of motion without causing pain [39, 40]. This study extends upon smaller scale exploratory research examining isokinetic knee extension and flexion strength recovery in 8 of the players examined without comparisons between graft types [41].

### Rehabilitation

Subjects followed a 6-month rehabilitation programme, which involved a 90-min session delivered on 6 days per week under the supervision of the same physical therapist, who was highly experienced in managing soccer players following ACL reconstruction.

Rehabilitative progression was determined for each subject using criteria in published guidelines [17, 42]. The rehabilitation programme was divided into phases based on the stage of tissue recovery and the ability of the knee joint to withstand loading demands (0–4 weeks, 5–8 weeks, 9–12 weeks, 13–18 weeks, and 19–24 weeks). During the first 3 days following surgery, focus was placed on range of motion (gradual progress with ~90° achieved by the end of week 1 and full knee flexion achieved in week 4 or 5) as well as managing pain and swelling. Subjects were on crutches for 2 weeks following surgery, after which full weight bearing was permitted as tolerated. Hydrotherapy was implemented for 2 weeks (e.g., deep water running, lunging, squatting, underwater cycling) 18 days after thread removal (thread removal was 16–18 days following surgery across subjects). Electrical stimulation (Compex SP 2.0; Compex Medical, Switzerland) was administered for 4 weeks after surgery to reduce the arthrogenic muscle inhibition effects of swelling, support the recovery of knee extensor strength, and activate the inhibited motoneurons. Movement complexity and speed of activities were systematically increased across isometric, isotonic, and isokinetic exercises. The rehabilitation protocol followed by subjects has been previously described in detail [41]. The load ratio for the quadriceps in seated knee extensions and hamstrings in seated leg curls differed between groups for the first 4 months of the rehabilitation programme (i.e., quadriceps:hamstrings of 60:40 for BPTB group and 40:60 for HT group). The quadriceps and hamstring muscles were equally loaded in both groups (i.e., 50:50) in months 5 and 6 of the rehabilitation programme.

### Isokinetic muscle strength

Knee extensor and flexor peak torques were measured at 3 months and 6 months following surgery using an isokinetic dynamometer (HUMAC-NORM, Model 770; Computer Sports Medicine Inc., Stoughton, MA, USA). The reliability (intraclass correlation coefficient = 0.82–0.93, typical error = 5.7–7.7 N · m) of the isokinetic dynamometer at an angular velocity of 60° · s^−1^ has been supported previously [43]. Each isokinetic strength test was performed in the concentric-concentric mode at an angular velocity of 60° · s^−1^, which has been suggested to be the most sensitive in capturing asymmetries between legs in peak torque measurements for the knee extensors and flexors in athletes who have undergone ACL reconstruction [44].

Subjects were seated in a chair with hips flexed to 90°. The trunk was fixed to the chair with two straps crossing the chest and a further strap positioned across the waist. Handles on either side of the chair were grasped during testing for consistent arm positioning. Straps were fastened across the thigh and malleoli on the tested leg to restrict any lateral movement, allowing only flexion and extension at the knee. The contralateral leg was fixed with the foot positioned behind an ankle stabilizer. Subjects performed five extension and flexion movements interspersed with 2 min of passive rest between movement types. Standardized instructions and verbal encouragement were given to each subject during testing. The contralateral leg was assessed first, followed by the injured leg using the same procedure with a 3-min passive resting period applied between legs. The highest peak torque (N · m) values recorded during knee extension and flexion were taken separately as outcome measures for each leg. LSI between legs and hamstrings:quadriceps ratio (H:Q) (concentric phase) within each leg were calculated as follows:

$$\text{LSI}=\frac{Injured\;peak\;torque}{Non-injured\;peak\;torque}\times 100\%$$

$$\text{H:Q}=\frac{Hamstring\;peak\;torque}{Quadriceps\;peak\;torque}$$

### Statistical analysis

An *a priori* power analysis using G*power software (version 3.1.9.4; Heinrich Heine University Düsseldorf, Düsseldorf, Germany) recommended a sample size of 16 players using an estimated effect magnitude based on research examining isokinetic muscle strength at 3 months and 6 months following ACL reconstruction in a subset of the present sample (p = 0.05, effect size [ES] = 0.30; power = 0.80) [41]. Normality of all data was confirmed using the Shapiro-Wilks test. Consequently, all data were reported as mean ± standard deviation (SD). Differences in outcome measures between BPTB graft and HT graft groups (graft effect), time points at 3 months and 6 months (time effect), injured and contralateral legs (leg effect), and muscle groups (muscle effect) were examined using separate 2 × 2 × 2 mixed analyses of variance (ANOVAs) with two within-subjects factors (either time and leg effects or time and muscle effects), and one between-subjects factor (graft effect). Partial eta-squared (η^2^) was utilized to indicate the ES for each mixed ANOVA, and was interpreted as [45]: *no effect* (≤ 0.04); *minimum effect* (0.05–0.25); *moderate effect* (0.26–0.64); or *strong effect* (> 0.65). Post-hoc comparisons between timepoints, between legs, or between muscle groups were examined using paired t-tests. Post-hoc comparisons between graft groups were examined using unpaired t-tests. Hedge’s g (with 95% confidence intervals [CI]) was also calculated to determine the ES for all post-hoc pairwise comparisons and was interpreted as [46]: *trivial* (< 0.20); *small* (0.20–0.49); *moderate* (0.50–0.79); or *large* (≥ 0.80). Statistical analyses were performed using IBM SPSS software (version 19; IBM Corp., Armonk, NY, USA). Statistical significance was accepted at p < 0.05.

## RESULTS

**FIG. 1:**
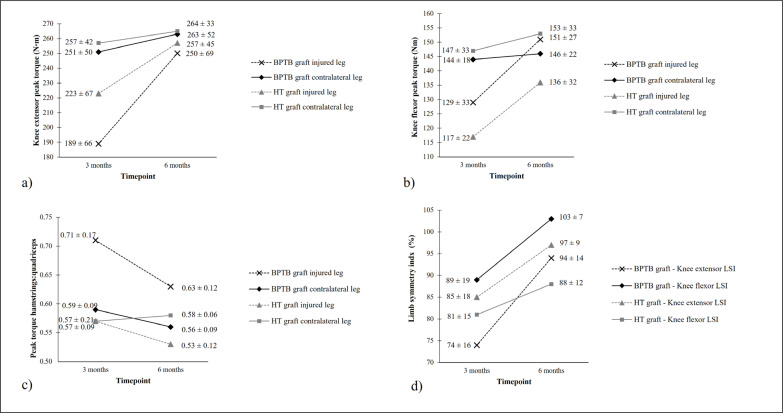
Mean ± standard deviation for (a) knee extensor peak torque, (b) knee flexor peak torque, (c) peak torque hamstrings:quadriceps ratio, and (d) limb symmetry index (LSI) in international male soccer players who underwent either a bone-patellar tendon-bone (BPTB) graft or hamstring tendon (HT) graft for anterior cruciate ligament reconstruction taken at 3 months and 6 months following surgery during the rehabilitation process.

**FIG. 2:**
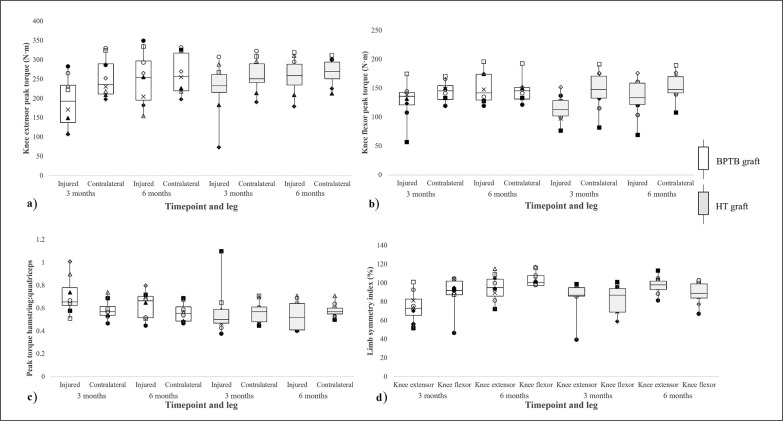
Individual data points for each subject and descriptive values for (a) knee extensor peak torque, (b) knee flexor peak torque, (c) peak torque hamstrings:quadriceps ratio, and (d) leg symmetry index in international male soccer players who underwent either a bone-patellar tendon-bone (BPTB) graft or hamstring tendon (HT) graft for anterior cruciate ligament reconstruction taken at 3 months and 6 months following surgery during the rehabilitation process. *Note*: Each marker represents a different subject. In the box plots, whiskers indicate the minimum and maximum values, the boundary of the box closest to zero indicates the 25^th^ percentile, the black line within the box indicates the median, and the boundary of the box farthest from zero indicates the 75^th^ percentile.

The results of each mixed ANOVA are presented in Table 1, with the mean ± SD for each outcome measure presented in Figure 1. Individual data, the median, minimum, maximum, and corresponding interquartile (25^th^ and 75^th^ percentiles) range in knee extensor peak torque, knee flexor peak torque, the H:Q for the injured and contralateral leg, as well as the LSI for the knee extensors and flexors at 3 months and 6 months following surgery are shown in Figure 2.

**TABLE 1:** Statistical outcomes from the mixed ANOVAs showing time (3 months vs. 6 months), leg (injured vs. contralateral legs) or muscle (knee flexor limb symmetry index vs. knee extensor limb symmetry index), graft (bone-patellar tendon-bone vs. hamstrings tendon grafts), and interaction effects for knee extensor peak torque, knee flexor peak torque, peak torque hamstrings:quadriceps ratio (H:Q), and limb symmetry index in international, male soccer players who underwent anterior cruciate ligament reconstruction.

Effect	Knee extensor peak torque		Knee flexor peak torque		Peak torque H:Q		Effect	Limb symmetry index	
	
	p	η2, *interpretation*	p	η2, *interpretation*	p	η2, *interpretation*		p	η2, *interpretation*
Time	< **0.001**	0.67, *strong*	< **0.001**	0.60, *moderate*	0.19	0.11, *minimum*	Time	< **0.001**	0.57, *moderate*
Time*Graft	0.14	0.14, *minimum*	0.82	0.00, *no effect*	0.45	0.04, *minimum*	Time*Graft	0.20	0.11, *minimum*
Leg	< **0.001**	0.55, *moderate*	**0.001**	0.55, *moderate*	0.24	0.09, *minimum*	Muscle	0.38	0.05, *minimum*
Leg*Graft	0.22	0.10, *minimum*	**0.02**	0.33, *moderate*	0.07	0.21, *minimum*	Muscle *Graft	**0.01**	0.37, *moderate*
Time*Leg	< **0.001**	0.64, *strong*	**0.04**	0.26, *moderate*	0.32	0.07, *minimum*	Time* Muscle	0.37	0.05, *minimum*
Graft	0.63	0.02, *no effect*	0.74	0.01, *no effect*	0.18	0.11, *minimum*	Graft	0.63	0.02, *no effect*
Time*Leg*Graft	0.16	0.13, *minimum*	0.63	0.02, *no effect*	0.91	0.00, *no effect*	Time*Muscle*Graft	0.85	0.00, *no effect*

*Note*: bolded p value indicates statistically significant effect at p < 0.05.

**FIG. 3:**
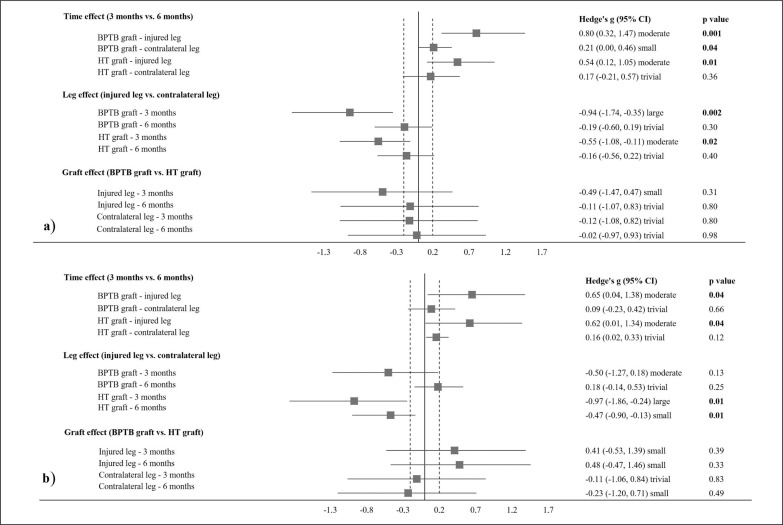
Statistical pairwise comparisons for (a) knee extensor peak torque and (b) knee flexor peak torque between timepoints, legs, and graft types in international male soccer players.

A 2 × 2 × 2 mixed ANOVA revealed a significant time*leg interaction (p < 0.001, η^2^ = 0.64), with subsequent significant main effects of time (p < 0.001, η^2^ = 0.67) and leg (p < 0.001, η^2^ = 0.55) in knee extensor peak torque. Follow-up comparisons (Figure 3a) revealed a significant *moderate* increase in knee extensor peak torque in the injured leg between 3 months and 6 months across both graft types (BPTB: p < 0.001, g = 0.80; HT: p = 0.01, g = 0.54), whereas a significantly *small* increase (BPTB: p = 0.04, g = 0.21) and non-significant *trivial* increase (HT: p = 0.36, g = 0.17) were observed in the contralateral leg between these time-points. In addition, significant *moderate–large* asymmetries in knee extensor peak torque between the injured and contralateral legs at 3 months for both graft types (BPTB: p = 0.002, g = -0.94; HT: p = 0.02, g = -0.55) were reduced to non-significant *trivial* differences between legs at 6 months (BPTB: p = 0.30, g = -0.19; HT: p = 0.40, g = -0.16).

A 2 × 2 × 2 mixed ANOVA revealed a significant time*leg interaction (p = 0.04, η^2^ = 0.26), with subsequent significant main effects of time (p < 0.001, η^2^ = 0.60) and leg (p < 0.001, η^2^ = 0.55) in knee flexor peak torque. Follow-up comparisons (Figure 3b) revealed a significant *moderate* increase in knee flexor peak torque in the injured leg from 3 months to 6 months across both graft types (BPTB: p = 0.04, g = 0.65; HT: p = 0.04, g = 0.62), with non-significant *trivial* changes evident in the contralateral leg between these time-points (BPTB: p = 0.66, g = 0.09; HT: p = 0.12, g = 0.16). A significant *large* asymmetry in knee flexor peak torque between the injured and contralateral legs at 3 months in the HT graft group (p = 0.01, g = -0.97) was reduced to a significant *small* difference between legs at 6 months (p = 0.01, g = -0.47). In addition, a non-significant *moderate* asymmetry in knee flexor peak torque between the injured and contralateral legs at 3 months in the BPTB graft group (p = 0.13, g = -0.50) was reduced to a non-significant *trivial* difference between legs at 6 months (p = 0.25, g = 0.18).

**FIG. 4:**
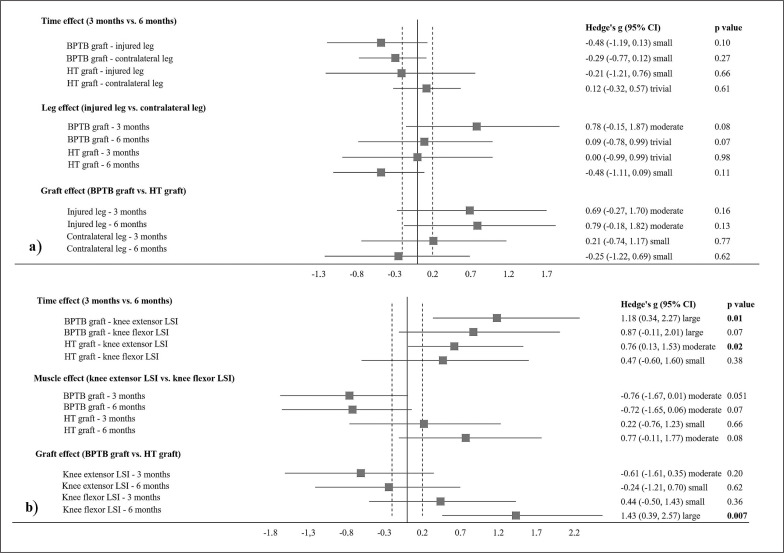
Statistical pairwise comparisons for (a) peak torque hamstrings:quadriceps ratio and (b) leg symmetry index (LSI) between timepoints, legs, muscles, and graft types in international male soccer players.

A 2 × 2 × 2 mixed ANOVA revealed non-significant interactions and main effects across all comparisons for H:Q, with non-significant *trivial–moderate* effects evident for all follow-up pairwise comparisons (Figure 4a).

A 2 × 2 × 2 mixed ANOVA revealed a significant muscle*graft interaction (p = 0.01, η^2^ = 0.37), with a subsequent significant main effect of time (p < 0.001, η^2^ = 0.57) in LSI. Follow-up comparisons (Figure 4b) revealed significant *moderate–large* increases in knee extensor LSI from 3 months to 6 months across both graft types (BPTB: p = 0.01, g = 1.18; HT: p = 0.02, g = 0.76), with non-significant *small–large* increases evident in knee flexor LSI between these timepoints for both graft types (BPTB: p = 0.07, g = 0.87; HT: p = 0.38, g = 0.47). Non-significant *small–moderate* differences were observed between knee extensor LSI and knee flexor LSI for both graft types at 3 months (BPTB: p = 0.05, g = -0.76; HT: p = 0.66, g = 0.22) and 6 months (BPTB: p = 0.07, g = -0.72; HT: p = 0.08, g = 0.77). There was a significant *large* difference in knee flexor LSI at 6 months in favour of the BPTB graft compared to the HT graft (p = 0.007, g = 1.43).

## DISCUSSION

The present results revealed that knee extensor peak torque was almost equivalent (BPTB: 0%; HT: 3%) to the contralateral leg regardless of the graft type used at 6 months following ACL reconstruction. Specifically, *moderate–large* asymmetries between legs in knee extensor peak torque at 3 months following reconstruction were reduced to *trivial* magnitudes at 6 months, with a desired difference of < 10% between legs across both graft types (BPTB: 6%; HT: 3%). Similarly, *moderate–large* asymmetries between legs in knee flexor peak torque at 3 months following reconstruction were reduced to *trivial–small* magnitudes across graft types at 6 months (BPTB: 0%; HT: 12%). However, despite the *trivial–small* asymmetries between legs in knee flexor peak torque at 6 months following reconstruction, graft-related comparisons revealed a *large* difference in knee flexor LSI with inferior strength recovery in the HT group compared to the BPTB group.

Comparisons across timepoints revealed *moderate* improvements in knee extensor peak torque in the injured leg, with LSI exceeding 90% for both graft types at 6 months following ACL reconstruction.

The improved knee extensor strength across time was further underpinned by *trivial* asymmetries between legs at 6 months following surgery for both graft types. Aligning with our results, several previous studies [22, 26–28, 30–32] have reported comparable knee extensor LSI across ACL reconstructions involving either BPTB or HT grafts. In contrast, some research has demonstrated significantly lower knee extensor LSI with a BPTB graft compared to a HT graft following ACL reconstruction [17, 18, 20, 21]. It has been postulated that neural factors such as a higher active motor threshold, reduced motor evoked potentials, brain plasticity, abnormal excitability of both spinal reflexive and corticospinal pathways, and damaged mechanoreceptors may underpin the prolonged weakness in the knee extensors sometimes observed after ACL reconstruction when using a BPTB graft [47]. Nevertheless, the bulk of existing research [22, 26–28, 30–32] and the novel findings we provided for a professional athlete sample (i.e., international-level, male soccer players) suggest that both graft types are sufficiently and equally effective in restoring knee extensor strength following ACL reconstruction. However, caution should be taken when making comparisons between our findings and those reported previously considering disparities in the timeframe adopted between ACL reconstruction and strength assessment (6 months [31], 9 months [26], 11 months [22], 12 months [32], 28 months [28], 29 months [27], and 36 months [30]) and angular velocities used during isokinetic dynamometry assessments (60° · s^−1^ [27, 28, 30, 32], 90° · s^−1^ (17), 120° · s^−1^ [26], 180° · s^−1^ [22, 31], and 300° · s^−1^ [22, 30, 31]). Specifically, longer rehabilitative periods prior to strength assessments may enable patients to better restore muscle strength regardless of the graft type used. Moreover, faster isokinetic angular velocities may produce larger strength deficits in assessments due to the greater recruitment of type II muscle fibres [25], which undergo more pronounced atrophy than type I muscle fibres following ACL reconstruction [48]. The limb symmetry in knee extensor strength observed in our study indicates that 6 months may be a sufficient recovery time-frame following a suitable rehabilitation programme to restore quadriceps strength in international male soccer players irrespective of using BPTB or HT grafts. In this regard, attaining suitable inter-limb knee extensor symmetry is important among soccer players given that this attribute has been shown to distinguish between different playing levels [14] and negatively correlate with change-of-direction and sprint performance [49], which are key movements performed during competition [50].

Like the knee extensors, we observed *moderate–large* improvements in knee flexor peak torque in the injured leg across timepoints; however, knee flexor strength reached an equivalent level to the contralateral leg only in the BPTB group, with a strength deficit of 12% apparent in the injured leg for the HT group at 6 months following ACL reconstruction. Similar to our findings, some researchers have observed greater asymmetries between legs in knee flexor strength among general patients [21], recreationally active patients [22], and non-professional athletes [26, 27] receiving a HT graft compared to a BPTB graft at various timepoints following reconstruction (i.e., 5–24 months) and using varied angular velocities during strength assessments (i.e., 60° · s^−1^ – 300 ° · s^−1^). Opposite to our findings, some studies have observed comparable knee flexor LSI between HT and BPTB graft types in recreationally active individuals [23], non-athletes [25], non-professional athletes [28], military cadets [30], and participants with varied activity levels [31] at various timepoints following reconstruction (i.e., 5–11 months) and using a range of angular velocities in strength assessments (60° · s^−1^ – 300° · s^−1^). However, despite some research showing comparable LSI between graft types following reconstruction (19, 21, 24, 26, 27), none of these studies demonstrated that the patient samples examined had reached the recommended strength recovery level (LSI > 90%) by 6 months with HT grafts during the rehabilitation process. In turn, the deficit in knee flexor strength with HT grafts compared to BPTB grafts consistently reported across various patient samples following ACL reconstruction may be partly attributed to chronic neuromuscular inhibition of the donor muscle [17], the slow regenerative capacity of the semitendinosus and gracilis tendons (up to 12–24 months following surgery) [21, 51], and the slower tendon-to-bone healing process (compared to the bone-to-bone tunnel healing evident in BPTB grafts) [52]. Although an almost acceptable knee flexor strength asymmetry between legs of 12% was reached in the HT group in our study, the apparent differences in strength recovery between graft types emphasize that clinicians should ensure that sufficient attention is devoted to hamstring exercises specifically targeting the semitendinosus and gracilis in players receiving a HT graft.

In addition to LSI, the conventional peak torque H:Q has been widely used to screen individuals at risk of sustaining ACL injuries, whereby decreased hamstring strength relative to the quadriceps has been identified as a potential risk factor for ACL injury in athletes [53]. In this regard, dominance in the quadriceps may increase anterior tibial translation and ACL loading, whereas concomitant hamstring coactivation provides dynamic joint stabilization that protects the knee during sport-related tasks [54]. The mean peak torque H:Q values we observed for both graft types at each timepoint were within the normative range (0.5 to 0.8) reported for professional male soccer players [55]. Also, data collected in our study revealed non-significant differences between timepoints, legs, and graft types. Specifically, non-significant changes in peak torque H:Q from 3 months to 6 months may be explained by the parallel strength improvements across both muscle groups we observed. The parallel improvement in knee flexor and extensor strength across the rehabilitation programme may be especially pronounced given the compromised muscle strength encountered after reconstructive surgery irrespective of the graft type used, which was evidenced by the lower LSI between legs we observed at 3 months compared to 6 months following ACL reconstruction. Considering the compromised muscle strength across both graft types, our results also suggest similar postoperative enhancements in H:Q with both BPTB and HT grafts. Furthermore, the peak torque H:Q did not differ between the injured and contralateral legs for players receiving either graft type, which has been proposed as suitable criteria, alongside adequate LSI values, in clearing players for return to unrestricted play [56].

It is important to acknowledge some key limitations of our study when interpreting the findings. First, isokinetic strength assessments immediately prior to surgery, in the early phase of rehabilitation, and taken across a longer time frame (> 6 months) were not conducted; they would have provided a better understanding concerning the effects of each graft type on knee muscle strength recovery and readiness to return to play among the examined subjects. Second, further functional measurements accompanying the strength assessments were not carried out in our study. In this regard, laxity tests [30], single leg hop tests [56], as well as closed and open kinetic chain rate of force development tests [16, 44] could be considered in conjunction with strength tests to indicate a more complete functional recovery status to support rehabilitative progression and determine readiness to play. Third, although isokinetic strength assessments were performed in a concentric:concentric contraction mode to prevent muscle strains, it should be acknowledged that ACL injuries may occur when the quadriceps are undergoing eccentric contraction in soccer [57], which emphasizes the potential importance of eccentric strength assessments during rehabilitation [58]. Fourth, while players having BPTB and HT grafts were comparable with respect to age, height, body mass, competition level, and pre-injury activity levels, a non-randomized design was adopted in our study.

## CONCLUSIONS

Our results demonstrate improvements in isokinetic strength, interlimb symmetry (knee extensor strength asymmetry: BPTB = 6%, HT = 3%, knee flexor strength asymmetry: BPTB = 0%, HT = 12%) and H:Q values across both graft types when following similar 6-month rehabilitation programmes. Since similar rehabilitation programmes were less effective at restoring knee flexor strength in subjects specifically receiving HT grafts, strength and conditioning professionals working with international male soccer players rehabilitating from ACL reconstruction after receiving a HT graft should pay adequate attention to delivering suitable hamstring exercises ensuring that optimal strength restoration occurs within a suitable timeframe for safe return to unrestricted play. Given that our study provides the first data concerning muscle strength recovery relative to graft type in international male soccer players, the provided data may help inform clinicians working with this population on expected outcomes between 3 and 6 months following ACL reconstruction.
